# A qualitative analysis exploring barriers and enablers to distribution, delivery, and access to COVID-19 vaccines in Botswana

**DOI:** 10.3389/frhs.2025.1609056

**Published:** 2025-11-18

**Authors:** John T. Tlhakanelo, John Ele-Ojo Ataguba, Vincent Pagiwa, Nankie Ramabu, Khutsafalo Kadimo, Grace Njeri Muriithi, Daniel Malik Achala, Elizabeth Naa Adukwei Adote, Chinyere Ojiugo Mbachu, Senait Alemayehu Beshah, Nyasha Masuka, Chijioke Osinachi Nwosu, James Akazili, Chikezie Ifeanyi, Dintle Molosiwa

**Affiliations:** 1 Department of Family Medicine and Public Health, Faculty of Medicine, University of Botswana, Gaborone, Botswana; 2 African Health Economics and Policy Association (AfHEA), Accra, Ghana; 3 Department of Community Health Sciences, Max Rady College of Medicine, Rady Faculty of Health Sciences, University of Manitoba, Winnipeg, MB, Canada; 4 Partnership for Economic Policy (PEP), Nairobi, Kenya; 5 School of Health Systems and Public Health, University of Pretoria, Pretoria, South Africa; 6 Okavango Research Institute, University of Botswana, Maun, Botswana; 7 Department of Library and Information Science, University of Botswana, Gaborone, Botswana; 8 Department of Community Medicine, University of Nigeria, Enugu, Nigeria; 9 Ethiopian Public Health Institute, Addis Ababa, Ethiopia; 10 Department of Public Health, Zimbabwe College of Public Health Physicians, Harare, Zimbabwe; 11 Department of Economics and Finance, University of the Free State, Bloemfontein, South Africa; 12 School of Public Health, C.K Tedam, University of Technology and Applied Sciences, Navrongo, Ghana; 13 BCEPS, University of Bergen, Bergen, Norway; 14 Health Systems and Development Research Group, Veritas University Abuja, Abuja, Nigeria; 15 School of Public Health, Walter Sisulu University, Mthatha, South Africa

**Keywords:** COVID-19 vaccines, equitable access, Botswana, distribution, delivery mechanisms, stakeholder engagement

## Abstract

**Introduction:**

The COVID-19 pandemic highlighted pre-existing weaknesses, revealing deep-rooted issues in infrastructure, access, and resource allocation that have long impeded African countries' ability to effectively meet population health needs. It also became evident during the pandemic that there were discrepancies in how vaccines were distributed, delivered and accessed in these countries. We aimed to identify vaccine distribution, service delivery processes and related barriers in Botswana to contextually explore practices that either enhance or hinder access and equity in vaccine distribution and delivery.

**Methods:**

We conducted in-depth interviews, using a semi-structured interview guide, with a purposive sample of 18 key informants, including public health sector officials, non-state actors, policy makers, regulatory bodies and other stakeholders. Interviews were audio-recorded and transcribed verbatim. Thematic analysis was conducted following a deductive approach according to the six-step analysis framework by Braun and Clarke: (i) familiarization with the data; (ii) generation of initial codes; (iii) searching for themes; (iv) reviewing themes; (v) refining and naming themes; and finally, (vi) producing the report. Steps i–iii were conducted by two researchers. Attention was given to aspects of credibility, dependability, and transferability of the findings through key strategies, including team data review, coding, consensus on themes and review of both secondary and grey literature on vaccine roll-out in the country.

**Results:**

Seven primary themes emerged from the findings. COVID-19 vaccine distribution and delivery in Botswana followed the existing primary health care system structures for routine vaccine delivery. Traditional mechanisms such as static public health facilities, private facilities, outreach campaigns, and mobile stops, were augmented through different roles played by stakeholders in the private sector, civil society organizations and non-governmental organizations. Religious and cultural norms were reported to affect vaccine uptake centered around smaller population groups that are historically known to be anti-vaccines. There is no deliberate gender and the disabled population programming for vaccine distribution and delivery in Botswana. The private sector improved access to vaccines by supporting supply chain logistics with transportation, especially to hard-to-reach areas.

**Discussions:**

Achieving equitable vaccine access involves not only logistical and infrastructural considerations, but also coordination and collaboration across several sectors, enhancing gender diversity and inclusivity in planning, coordination, and decision making and implementation of strategies tailored to the needs of a wide range of vulnerable population groups.

## 1 Introduction

The COVID-19 pandemic underscored global disparities in vaccine distribution and access, prompting renewed discussions on strengthening vaccine research and development capabilities—particularly in low and middle-income countries (LMICs) (1). In Africa, vaccine delivery has been characterized by persistent inequity and inefficiency arising from both health system and population factors (2, 3). The challenges become more pronounced during the COVID-19 pandemic, with various studies revealing challenges including inadequate health financing, logistical hurdles, fragmented care, ineffective cold chain management and vaccine hesitancy (4–6). Nkole et al. (2023) observed that these challenges were further exacerbated by top-down implementation approach driven by international agendas, which often restricted in-country proper administration of the vaccine distribution and delivery (5). The long-standing structural barriers caused by inequitable global demand, high procurement costs, weak manufacturing capabilities and inefficient global allocation mechanisms for vaccine distribution also exacerbated delayed and unequal access to available vaccines for Africa's population. Despite these barriers, other studies suggested enablers that contributed to more equitable vaccine distribution and delivery. These facilitators include robust community engagement, precisely community-led monitoring (5), proper cold chain infrastructure and transportation systems. Effective documentation and monitoring mechanisms, continuous research, cross-sectoral collaboration, and culturally tailored strategies with localised approaches have also been shown to enhance vaccine distribution in resource-limited settings (4, 5, 7–9). Prior to and throughout the COVID-19 pandemic, vaccine distribution and access continued to be shaped and influenced by high uncertainty in demand and supply, political dynamics and deeply rooted equity concerns pointing to glaring disparities between and within countries (10). Limited distribution and access to vaccines in LMICs due to global demand and supply constraints, structural barriers around local manufacturing meant that countries with insufficient finances and logistical capacity also saw increased disparities among their populations—affecting especially vulnerable populations (11). Only when countries work together with their efforts grounded in ethical and procedural principles can they achieve equitable allocation of vaccine, maximize public health benefits—explicitly focusing on mitigating health inequities. As countries move towards building resilient health systems (12) post-COVID 19, it is imperative that policy makers, and practitioners develop effective and evidence-based frameworks that systematically evaluate and improve vaccine distribution and access. Such frameworks should recognize the multiplicity of challenges posed by complexities in supply chain, ethical considerations towards fair allocation, diverse socio-economic and cultural barriers that present differently across diverse settings/regions of the world. This understanding, coupled with the above cited approaches, should drive adaptable strategies, inform operational decisions that prioritize equity and maximize vaccine distribution and access, if effective response to public health threats is to be achieved by all.

High income countries such as Germany, Belgium and Australia have demonstrated that strategically placing the community at the center of locally-led COVID-19 vaccine distribution and delivery, ensuring seamless information flow among health professionals, and leveraging broad media communication are enablers of equitable vaccine delivery (13, 14). These insights have underscored the urgent need to re-imagine vaccine distribution and delivery by boosting domestic vaccine production in Africa, as a pivotal strategy to address existing inequities and foster a more equitable global health system (15, 16). Africa possesses the capacity to produce its vaccines, vaccine raw materials, diagnostics, and other medical supplies. However, realising this potential requires strategic alignment of Africa's investment priorities and the promotion of public-private partnerships or product development partnerships, to further expand capacity for research and development of vaccines across the continent (17–19).

This study focuses on Botswana, highlighting the country's experience with COVID-19 vaccine distribution and delivery in the context of enhancing access and equity. Like many health systems in Africa, Botswana's health system aims to improve vaccine access and uptake, particularly during public health emergencies such as the COVID-19 pandemic, while promoting equitable distribution and strengthening delivery mechanisms across all levels and population groups. By leveraging its public healthcare infrastructure and longstanding public health strategies, Botswana achieved notable vaccination coverage, with over 70% of the population receiving at least one dose by 2023, significantly curbing the spread of the virus and mitigating its impact on national health outcomes (20). We aimed to identify vaccine distribution, service delivery processes and related barriers in Botswana to contextually explore practices that either enhance or hinder access and equity in vaccine distribution and delivery. The assessment of these enablers and barriers will provide the critical information for the government's vaccine investment decisions, vaccine development and effective distribution amongst the population, particularly among vulnerable groups. More specifically, the study sought to provide nuanced issues, exploring mechanisms and or practices that can help in the mitigation of vaccine inequities and contribute to national and international literature on equitable vaccine distribution mechanisms. Finally, this study was intended to investigate the role of civil society organisations, non-governmental bodies, and the private sector in improving vaccine delivery and distribution in Botswana.

## 2 Materials and methods

### 2.1 Study design

We conducted a qualitative study, in which data were collected through in-depth interviews using a semi-structured interview guide. A total of 18 key informants were purposively sampled, representing 72 percent of the initial target sample of 25 identified as potential participants for the study. The study sample was carefully identified to represent various organizational categories related to COVID-19 distribution and delivery, including but not limited to, surveillance and pharmaco-vigilance, planning and coordination, vaccine cold chain, logistics and infrastructure, vaccines and pharmaceuticals supply, regulatory and protection roles, budgeting and finance, Expanded Program on Immunization (EPI), disease surveillance, Risk Communication and Community Engagement (RCCE), administration, operational research and vulnerable groups services. Participants were sampled from various entities that include the Ministry of Health (Expanded Program of Immunization, Central Medical Stores, Botswana Public Health Institute, Health Services), Botswana Medicine's Regulatory Authority (BOMRA), bilateral organisations such as World Health Organization (WHO), United Nations Children's Fund (UNICEF), United nations Population Fund (UNFPA), non-governmental organizations, as well as community-based organisations based in Gaborone (capital city) and involved in the COVID-19 vaccine roll-out in Botswana. Participants were approached physically at their organizations and followed up with phone calls and emails to improve response. Some of those who declined participation were generally non-responsive to repeaeted follow ups with no explicit reasons, while some reported lack of time and non-authorization to participate in the study from their supervisors.

The qualitative approach was considered appropriate for this study because we were interested in exploring the “how” and “why” questions in vaccine access and equity. Qualitative research seeks to understand the complex relationship between the personal and social meanings, individuals and cultural practices, and the material environment or context—giving insights into why and how things work the way they do (21). The research team had extensive expertise in qualitative research and healthcare quality in general practice. The interview guide is included as a supplement (Supplementary File S1).

**Inclusion criteria**: Key informants included people who were directly involved in the COVID-19 vaccines procurement, delivery and distribution process in Botswana, worked for government, NGOs, or the private sector in any of the diverse selected organizational categories of interest.

**Exclusion criteria**: We excluded officials who were not directly involved in COVID-19 vaccine distribution and delivery and those whose workplaces were based outside the capital city, Gaborone.

### 2.2 Data collection

Data collection took place between May and August 2024. It entailed face-to-face in-depth interviews with 18 key informants who were involved in the country's vaccine roll-out and were available to be interviewed. All interviews were conducted in English, in private locations preferred by participants, by experienced qualitative data collectors, and lasted between 30 and 90 min. The interviews were audio-recorded. Experienced interviewers were recruited and trained over two days on the study protocol, research ethics, and interview skills by the researchers.

Concept saturation was used to determine the final sample size. Data saturation was assessed during the process of conducting the interviews—where the interviewers met to discuss the interviews, focusing on each question. The discussions included each interviewer highlighting preliminary themes emerging for each question. Two rounds of discussion meetings were held during the data collection period and saturation was determined when it was agreed that the new data were not adding any new/additional themes.

A one-day workshop to validate findings was held following analysis, where results the study findings were presented and discussed with participantskey informants. Although no additional themes were derived from the workshops deliberations, further recommended interventions on minimizing barriers to equitable vaccine delivery and distribution were recorded and incooporated into the study findings. The workshop was attended by government nine (9) original participants while others sent representatives and other officials who were not part of the study sample but were involved in COVID-19 vaccine distribution and delivery. These included experts from the University of Botswana, the Institution of Development mManagement, Ministry of Health, Botswana Medicines Regulatory Authority, World Health Organisation and the Botswana Red Cross.

### 2.3 Data analysis

All audio recordings were transcribed verbatim and analyzed using deductive thematic analysis, where a codebook was developed (by NR) using predefined themes from interview guide questions. Both NR & DM are experienced researchers and they conducted the analysis both manually and with the aid of qualitative data analysis software. In addition to data saturation established during data collection, saturation was also established during data analysis following the concept of theoretical/concept saturation—where reading through and coding the 18 transcripts, no new codes and themes were evident from the data as agreed between the two analysts (NR & DM). The two analysts read through the transcripts—each selecting and independently reading 3 transcripts and discussing an initial codebook developed by NR. The two analysts iteratively reviewed all the transcripts manually to note the themes emerging from the data. An additional analysis was done using Nvivo software to further develop rich and detailed descriptions of the themes and cover the dimensions of the agreed upon codes. The software efficiently segmented the textual data, allowing us to examine similarities and differences and group the data according to our research questions using its advanced tools for data management and visualization. By manually reviewing and interpreting the data, we ensured that the nuances and context of the information were preserved and accurately represented. This dual approach allowed us to leverage the strengths of both methods—thus providing a balanced and thorough exploration of the data, enhancing the credibility and richness of our findings while mitigating potential limitations inherent in relying solely on either method (22). Fully aware of our positionality in healthcare delivery (where both analysts are primarily academic researchers), we compared and mapped the relationship between the themes and were able to establish a comprehensive exploratory framework of vaccine distribution and delivery mechanisms as experienced in Botswana during the COVID-19 pandemic. We reflexively examined how our experiences with the healthcare delivery systems might influence our data interpretation by engaging in extended dialogue throughout the analysis.

Thematic analysis, as described by Kieger and Varpio (23), was conducted following a deductive approach guided by the six-step analysis procedure by Braun and Clarke (2006: 16–23): (i) familiarization with the data; (ii) generation of initial codes; (iii) searching for themes; (iv) reviewing themes; (v) refining and naming themes; and finally, (vi) producing the report (24). Steps i–iii were conducted by researchers DM and NR. Attention was given to aspects of credibility, dependability, and transferability of the findings through several key strategies, including team data review, coding, and consensus on themes and review of both secondary and grey literature on vaccine roll-out in the country (25).

#### 2.3.1 Trustworthiness of this study

Data collection rigor was obtained by ensuring representation of participants from COVID-19 vaccine distribution and delivery stakeholders across sectors that represent government, non-governmental, private and community-based organisations. A structured interview guide was used, but still allowed for conversational flow. Data collection and analysis were conducted by trained and experienced interviewers. The researchers ensured consistent coding in line with Braun and Clarke (2006) through discussions among the experienced the coding team to compliment and challenge each other's perspectives, enriching and refining the analysis (24, 26, 27).

#### 2.3.2 Researcher reflexivity & insider knowledge

Since this was a qualitative research study that relied on nuanced judgments, it required researcher reflexivity. According to Sabnis & Wolgemuth (2023, pg.242), reflexivity is defined as *a set of continuous, collaborative, and multifaceted practices through which researchers self-consciously critique, appraise, and evaluate their subjectivity and context that influence the research process*. This approach acknowledges subjectivity as a valuable lens through which qualitative inquiry is shaped and appreciated. Taking into account the aforementioned definition of reflexivity, researchers took this opportunity to acknowledge and capitalize on their subjectivity in the investigation as an integral part of this investigation (28, 29). The research team comprised health professionals who had previously served as practitioners and currently serve as policy makers and trainers within Botswana's healthcare system. Crucial to this reflexive practice was the researchers' role in implementing equitable access to COVID-19 vaccines in-country. This shared investigator-participant experience provided a rich contextual understanding and insider status that enhanced the depth and authenticity of the data collected.

Additionally, being natives of Botswana, the researchers possessed intimate knowledge of the local culture and language, which granted access to nuanced information that facilitated rapport with participants, an advantage that wouldn't have been possible for an outsider. However, this epistemological stance also introduced limitations. One in particular is familiarity with systemic barriers, which could lead to their normalization, potentially obscuring critical insights that an outsider might more readily identify. Moreover, the research team's insider status meant they were intimately linked to the research process from study design to interpretation of the findings. While this closeness could be perceived as a source of bias (30), it was openly acknowledged and addressed through ethical reflexive practices. One such practice was hosting a validation workshop with stakeholders who participated in the research. This collaborative forum served to corroborate and enrich the findings, ensuring they were a true representation of the data shared by participants (31).

By the researchers embracing reflexivity, they aligned with qualitative standards that value ethical engagement, contextual sensitivity and transparency. Essentially, the research team's positionality was not a threat to rigor but a resource that strengthened the credibility and relevance of the study's outcomes (28, 29, 32).

## 3 Results

The study interviewed a total of 18 participants, with a median age of 47 years, half of whom were males. The majority the participants held a master's degree and 4 years' experience working in vaccine-related services. Table 1 provides a descriptive summary of the study key informants.

**Table 1:** Selected demographic characteristics and organizational categories of key informants.

Organizational category	Age				Sex		Highest level of education			Years in vaccine role				**Total**
	≤40	41–45	46–50	>50	Male	Female	Diploma	Degree	Masters	≤3	4	5	≥6	
Surveillance & Pharmaco-vigilance			x	x	xx			xx			x		x	2
Planning & Coordination	xx		x		x	xx			xxx	x	xx			3
Vaccine, cold chain, logistics & infrastructure			x			x	x						x	1
Vaccines & Pharmaceuticals supply		x				x			x		x			1
Regulatory				x		x			x		x			1
Budgeting & Finance				x		x			x			x		1
EPI Surveillance	x			x	x	x			xx	x	x			2
RCCE	x				x				x		x			1
Protection	x				x				x	x				1
Administration		x		x	x	x			xx	x	x			2
Operational research	x				x			x					x	1
Vulnerable groups services			x	x	x	x		xx		x	x			2
TOTAL (%)	6 (33%)	2 (11%)	4 (22%)	6 (33%)	9 (50%)	9 (50%)	1 (6%)	5 (28%)	12 (67%)	5 (28%)	9 (50%)	1 (6%)	3 (17%)	18(100%)

The following results are organized around the different elements that were explored through in-depth interviews with emerging themes indicated in sub-headings. The results of this study reveal the critical role of Botswana's longstanding principle in health service delivery, which is multisectoral coordination. The results also reveal the importance of government leadership supported by non-state actors, including development partners, and how leveraging existing infrastructure and community networks can help enhance vaccine delivery and equitable access. Most notably, the results show the multifaceted enablers and barriers embedded within the logistics, social dynamics, and stakeholder engagement processes. Insights gleaned through interviews with study participants provide critical lessons for health system strengthening to promote responsive, inclusive, and sustainable immunization strategies, prioritizing especially the needs of vulnerable populations that are often neglected.

### 3.1 Roles and responsibilities

#### 3.1.1 Multisectoral involvement and complementarity of roles of governmental and non-governmental actors

The complexity of Botswana's COVID-19 vaccine roll-out was clearly illustrated in this study. First the multiple actors who played different and complementary role, led by the Ministry of Health, created networks and coalition building by creating committees that enabled coordination for vaccination roll-out plan. This coalition-building approach, where each actor had distinct but complementary role was essential to navigate the intricate demands of cold-chain management, procurement fluctuations, and shifting target populations. However, equitable access remained a persistent challenge, particularly for geographically and socially marginalized populations, as reported in subsequent sections, underscoring the necessity for adaptive strategies that integrate community-level engagement and data-driven decision-making, especially under rapid change change and uncertainity. Various stakeholders, including public health organizations and development partners like the World Health Organization (WHO), United Nations Children's Emergency Fund (UNICEF), United Nations Development Plan (UNDP), and United States Agency for International Development (USAID), supported Botswana is her vaccine delivery. This collaboration served not only as a mechanism for overcoming operational barriers impacting procurement and distribution, but also as a platform to address equity gaps, highlighting the critical role of governance, stakeholder synergy, and system capacity strengthening in achieving inclusive vaccine coverage. (Table 2, Rows 1–2). Healthcare providers, including doctors, nurses, and pharmacists, and other local non-governmental organizations, executed the actual administration of vaccines, ensuring that they reached individuals efficiently and safely (Table 2, Row 3). Civil society organizations and other non-governmental organizations played a significant role in demand creation, community mobilization, addressing vaccine hesitancy, disseminating accurate information, and reaching underserved populations. Logistical companies and supply chain managers ensured the timely delivery of vaccines, while technology platforms facilitated registration, scheduling, and tracking of vaccinations. Collectively, these diverse roles and responsibilities, were essential in managing the country's vaccination roll-out plan. While a strong partnership with civil society and non-governmental organizations were was emphasized by participants, partnership with the private sector was reportedly weak owing to a lack of regulations on the private sector's social responsibility in the country: (Table 2, Row 4).

**Table 2:** Barriers and enablers to distribution, delivery, and access to COVID-19 vaccines from the perspectives of 18 key informants in Gaborone, Botswana.

Row	Theme	Subtheme	Key Informant Interview (KII)	Representation Quote
1	Roles & Responsibilities	Multisectoral Involvement & complementarity of roles	KII 3, Female	*We were relying on the World Health Organization … to determine the standards that we were using.*
2			KII 15, Female	*UNICEF was the designated institution for procurement coordination. Secondly, we supported the Ministry of Health to develop the national plan for devolution of the vaccines into the country. We also supported risk communication and engagement around COVID-19 vaccination*
3			KII 1, Female	*We were tasked with probably the primary thing, being vaccinating people in their communities in ten districts (out of 18 at the time) through mobile teams encompassing two nurses and two data clerks … to try and assist hard-to-reach communities and improve coverage and equity*
4		Interorganizational relationships	KII 8, Male	*Do not plan alone and then bring them at the end. Let us involve them from the beginning. When you formulate objectives, when you formulate programs, you should make sure that you bring in everyone to be part of the process, the planning process, the implementing process, the monitoring and the reporting process. Let us involve everyone from the beginning. And we used to just plan and then just give them a product and say, let us implement. And we will in most cases face challenges because they were not involved from the inception of the program. So when we started, they were a little behind. But when things got worse, we brought them in…*
5	Distribution and Delivery Mechanisms	Leveraging existing infrastructure and human resources	KII 11, Male	*…similar in a way to the expanded program on immunization.*
6			KII 12, Female	*…Central Medical Stores (CMS) remained the core player.*
7			KII 18, Male	*… there is a system in place that they [CMS] use to ensure that the vaccine reaches every level of implementation or facility … So, the system is such that the district orders from CMS and they engage (parastatal) courier service that is offered by Botswana Post to ensure that the vaccines are delivered at the district warehouses.*
8			KII 12, Female	*temporary vaccination sites such as going to workplaces … innovative ones like drive-throughs and really anywhere we could get people vaccinated.*
9			KII 8, Male	*a logistics team, in terms of looking at the targets and then procuring the right number of vaccines to reach the target. And the logistics team was working with us, of course, to set the targets and working with Central Medical Stores for distribution of vaccines. And vaccines were distributed, remember I talked about the phases where the vaccines were distributed according to the targets, meaning that they were looking at the district with high volumes and making sure that high volume vaccines go to those districts and districts with lower volumes were rationalizing vaccines to ensure that there is equitable distribution across the districts, and we were using district targets to distribute vaccines. And the vaccines were managed by pharmacists in every district, working with M&E officers in all those districts.*
10			KII 5, Male	*it was quite a comprehensive deployment or exercise. Um, we tried to involve everyone as best as we can, as you know, you might know there was some subcommittees which were formed just to assist with distribution and service delivery. And then those subcommittees, we tried to capture or cater for everyone who might be involved in the community.*
11			KII 7, Female	*As time progressed, the Ministry and other stakeholders procured more storage capacity*
12		Challenges to distribution and delivery mechanisms	KII 11, Male	*there are issues of roads, sometimes even when you are in a district, to reach all the corners of that district.*
13			KII 8, Male	*Basically, it was transport from the holding centers to the vaccination sites where we found some health workers using their own vehicles to transport vaccines. You know vaccines are transported in a very special way.*
14	Demand Creation		KII 13, Female	“Because we are a community-based entity, we create awareness amongst communities about the vaccines, about the side effects and we also try to alleviate and correct, you know, misconceptions that are in the community … *We were working very closely with partners who are vaccinating and then we had multiple projects where we were creating demand”*
12			KII 13, Female	“… *And not just civil society, there are structures in the community that are there, and you know, people listen better to someone they interact with* *..* *structures like the VHC, structures like VDC”*
13	Societal and cultural norms	Norms affecting vaccine uptake	KII 18, Female	*“yes, we still have pockets of our communities that are not very compliant, maybe because of their cultural beliefs or religious beliefs. We cannot say the significance in terms of numbers, but we know that these populations are there in Botswana and they are not confined to one place” (KII 18, Female)*
14			KII 8, Male	*When we started there was little vaccine hesitancy because we started when we already had cases. And remember we started around March and in winter more cases and deaths started affecting the country. And that reduced the vaccine hesitancy. So, we did not have that much cultural issues to vaccine hesitancy. Even the people that we know, like the Zezurus tribe, who do not vaccinate mostly their children, some of them did vaccinate because they could see that this thing was serious.*
15			KII 8, Male	*According to our data, the group that did not vaccinate much and we are yet to find out, are the adolescents, both men and women, boys and girls, they did not vaccinate “… we had targeted certain age group and when you look at our target for adolescents, we only vaccinated about 40% of those adolescents. The adults vaccinated very well. The mid-adults vaccinated very well. Of course, the other groups, school-going groups, vaccinated very well because they were captured because they were at school, the students.”*
16	Gender, vulnerable populations & equity	Vaccine distribution & delivery approaches	KII 15, Female	*COVID-19 was not just affecting women or men. So, I do not think there needed to be a gender lens..*
17			KII 2, Female	*First of all, a person would say the country tried by all means to reach out to Batswana in general just like we know, we are also told that this is for every Motswana. When something is done, we are told to believe that it is for us, now the issue comes with accessibility, that is, are all Batswana able to access, because as for when I speak in our perspective as persons with disabilities, we were left behind.*
18		Gender mainstreaming	KII 9, Male	*“in terms of gender-wise … more females got vaccines compared to their counterpart” (KII 9, Male)*
19			KII 12, Female	… *as a country, gender is not a thing that we are always saying this is for males, this is for females, and we did not even see changes in uptake, like differences in uptakes of vaccines in gender. It was not that big.*
20			KII 11, Male	*The issue of gender, I think sometimes we do not really appreciate because we do not know what is, what are the gender, because in some countries I understand that there are some serious gender issues because of their cultures, but in our cultures and our communities, whether it's a man or a woman, the messaging is usually not very different, the access is not usually very different. The girl child they sit in the same class with the boy. There is usually, as far as I know, there is usually very little, minimal difference between the genders in terms of access to vaccines, to information, to everything.*
21			KII 18, Female	*I think this question, I have really said that for Botswana, we don't really disaggregate. There is no service that we say for females or males, I mean, when you speak about vaccinations or immunizations. So even the message will come out just like that, open to everyone. Then it's an Individual decision to say how do I do, how do I take this message as a man, am I coming out to go and get the service that is being offered. So, it is really tried by all means to balance and make sure that everybody has an opportunity to benefit.*
22		Composition of coordination structures and committees	KII 8, Male	*In any organization, we should make sure that there is that involvement of women. But there will be those areas where you find the predominantly led by women* vs. *some areas. For instance, in the army, you will find that in the army, most prominent gender is males, because we started adding the females late.*
23		Vulnerable populations	KII 12, Female	*So vulnerable, I think the way to say someone is vulnerable is to say are they more likely to get COVID-19? So that is one way of saying you are vulnerable. So as far as COVID-19 goes, we have three main groups. One is people who are health care workers, because they come into contact with many people, sick people, COVID-19 patients. We have people who have comorbidities. The reason for comorbidities is if they get COVID-19, they are more likely to have complications, they are more likely to have severe disease, they are more likely to die. Elderly people, the same reasons. If they get COVID-19, they are more likely to have severe disease, they are more likely to have complications, they are more likely to die. So those are the vulnerable populations.*
24			KII 10, Female	*…we have a system which is working, it is very broad and fortunately you find it in very remote areas.*
25			KII 12, Female	*I think logistics need to come up with a good plan of how our society is structured so that when vaccines are distributed, you do not leave anyone behind. I talked about the farm workers in the farms. When the logisticians plan, they need to make sure that they come up with ways of reaching those people that are in the hard-to-reach areas.*
26	Stakeholder engagement	Multisectoral approach to stakeholder engagement	KII 1, Female	*This was an emergency response. So, because it was an emergency response, the involvement was at different stages. The initial involvement probably was multi-sectoral and a lot of departments were involved, military, police, you know, everyone in public sector and private sector.*
27			KII 4, Male	*“When you look at the number of stakeholders that were involved, and how they were coordinated, and how they delivered… that is a typical example of what we ought to do and improve on”.*
28		Coordinated systems of vaccine delivery and distribution	KII 4, Male	*The most important thing is to bring people on board early on, to show people the strategy and the plan and to see who will fit where and how, rather than to develop all of this and then you come and start the implementing*
29		Gender equity in stakeholder engagement	KII 13, Female	*I believe it [gender representation] is very important because women could understand women's issues better and could project the voice of women better… male, female and other gender identities should be represented so that they voice out their needs.*
30			KII 5, Male	*When you talk about issues of gender equity in the context of Botswana, I do not really know how to answer you in that. Because I would say in Botswana everyone is given an equal share. You cannot really say maybe women are disadvantaged because they are women. Everyone who has access to vaccines, they can always access it [vaccine] and they would not be having any problems and stuff.*
31		Challenges in stakeholder engagement	KII 2, Female	*When something is done we are told to believe that it is for us, now the issue comes with accessibility, that is are all Batswana able to access, because I speak in our perspective as persons with disabilities … we were left behind, because mostly you talk of the information, well, it was all over, on the radio, on TV but then again even the task team itself, I think it was supposed to have some reps from those persons with disabilities*
32	Public Private Partnership		KII 8, Female	*There are a lot of discussions around that [local vaccine manufacturing]. For Botswana we already have the Botswana Vaccine Institute, though we are looking at vaccines related to animal health. But these are systems in place that we can ride on to ensure that as a country we also start manufacturing vaccines for ourselves. The only disadvantage for Botswana at times is our population, so you will find that it's not very easy to break through the market. But our leaderships have been using every opportunity they get to advocate for such.*

Healthcare providers, including doctors, nurses, pharmacists, and other local non-governmental organizations, executed the actual administration of vaccines, ensuring that they reached individuals efficiently and safely (Table 2, Row 3). Civil society organizations and other non-governmental organizations played a significant role in demand creation, community mobilization, addressing vaccine hesitancy, disseminating accurate information, and reaching underserved populations.

**Figure 1:**
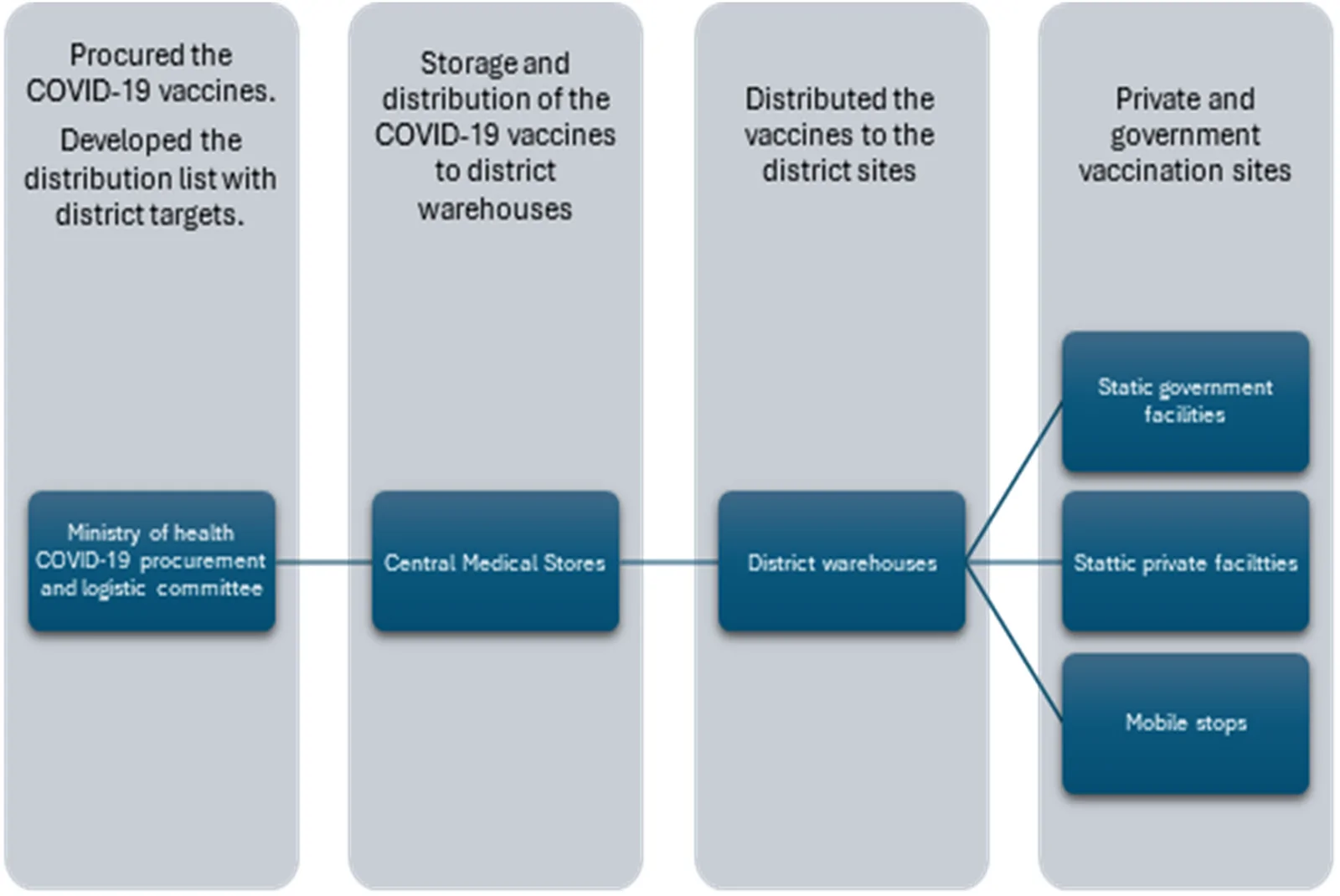
Vaccine distribution and service delivery pathway in Botswana.

The following infographic, Figure 1, summarises the role of stakeholders in vaccine distribution and delivery in Botswana.

#### 3.1.2 Clarity of roles and responsibilities

Logistical companies and supply chain managers ensured the timely delivery of vaccines, while technology platforms facilitated registration, scheduling, and tracking of vaccinations. Collectively, these diverse roles and responsibilities were essential in managing the country's vaccination roll-out plan. While a strong partnership with civil society and non-governmental organizations was emphasized by participants, partnerships with the private sector was reportedly weak owing to a lack of regulations on the private sector's social responsibility in the country: (Table 2, Row 4).

#### 3.1.3 Relationship between and among government and non-government actors

While a strong partnership with civil society and non-governmental organizations was emphasized by participants, partnership with the private sector was reportedly weak owing to a lack of regulations on the private sector's social responsibility in the country (Table 2, Row 4).

### 3.2 Distribution and delivery mechanisms

#### 3.2.1 Leveraging existing infrastructure and distribution and delivery mechanisms

Achieving desirable COVID-19 vaccine coverage levels in the country was a result of a well-coordinated and multi-faceted approach that leveraged on and was “similar in a way to the expanded program on immunization” (Table 2, Row 5). (KII 11—Male) routine distribution and delivery mechanisms. For vaccine procurement and distribution Central Medical Stores played a key role and used the system that already existed (Table 2, Rows 5–7). Leveraging an existing and central coordinating entity (CMS) to oversee vaccine distribution efforts ensured that key logistics, distribution, and vaccine administration were in line with national distribution and service delivery goals for vaccine roll-out. This was further strengthened by re-deploying existing human resources, supported by and working closely with other non-state actors to ensure an effective and comprehensive approach to vaccine roll-out (Table 2, Row 10). Participants also noted that “*As time progressed, the Ministry and other stakeholders procured more storage capacity” (Table 2, Row 11),* to strengthen the country's cold chain infrastructure (Table 2, Row 11). This included equipping health facilities with refrigerators and freezers capable of handling vaccines requiring ultra-cold storage.

Thus, a clear pathway for vaccine distribution and service delivery in Botswana could be traced and summarized (Figure 1).

#### 3.2.2 Challenges to distribution and delivery mechanisms

Although vaccine distribution and delivery mechanisms in the country hinged on existing and functional structures, various challenges were encountered. The storage of vaccines was restricted to bigger health facilities, which had the cold chain facilities and space to store vaccines, such as district hospitals and private hospitals. This meant that smaller facilities would collect vaccines daily, then return the surplus for the day to the storage facilities. Botswana is a vast country with many small villages and settlements that can also be physically hard-to-reach and “*there are issues of roads, sometimes even when you are in a district, to reach all the corners of that district” (Table 2, Row12).* Some of the challenges encountered centered around transport issues, including an insufficient number of vehicles to transport vaccines and healthcare workers from district warehouses to reach the last mile. Shortage of health care personnel was also indicated to have caused critical delays in vaccine distribution and service delivery. Lack of reliable transport also meant compromising on the vaccine cold chain, as ambulances and personal cars were sometimes used with very little attention given to maintaining the cold chain. (Table 2, Row 13).

The other challenge experienced by healthcare providers was around the opening of vaccine vials, where they required that there be a certain number of people present to receive the vaccine before opening the vial. Sometimes this meant making people wait longer, while in other instances people were requested to come the next day.

COVID-19 vaccine distribution and service delivery were facilitated through robust data collection to monitor the disease incidence among the population while also ensuring target coverage levels are reached and adverse events following immunization are monitored. Various surveillance efforts, including both passive and active surveillance for influenza-like illness, were instituted through existing Ministry of Health units, both within the EPI unit and through the Integrated Disease Surveillance and Response Unit—coordinated through the established committees that also supported data management and reporting. Although surveillance was reported to be generally good by participants, it was also indicated that it could be strengthened to improve monitoring and reporting of the COVID-19 vaccine distribution and delivery (Table 2, Row 9).

### 3.3 Demand creation

Extensive public awareness campaigns to educate citizens about the benefits of vaccination were launched. These campaigns used various avenues and media channels, including radio, television, and social media, *Kgotla* (Community Customary Court) meetings, door-to-door, as well as health facility morning talks to reach diverse populations and were supported mainly through civil society and other non-governmental organizations such as the Red Cross (Table 2, Row 14).

The success of demand creation activities was attributed to the political will behind the COVID-19 vaccination program, which also included leadership at the community level. Botswana has a long history of working with community structures to mobilize and promote health service utilization among community members. Community leaders and other local influencers played a critical role in advocacy during the COVID-19 vaccine roll-out. Engaging these trusted figures helped build vaccine confidence and encouraged higher uptake as information was able to reach people at the grassroots level (Table 2, Row 12).

### 3.4 Societal and cultural norms

#### 3.4.1 Norms affecting vaccine uptake

Societal and cultural norms negatively affecting vaccine uptake were not as widespread. On the contrary,fear of seeing relatives and families dying was reported as a motivator to get vaccinated among the vulnerable population of older age groups, frontline workers in particular, and healthcare professionals. There is an entrenched understanding that “*yes, we still have pockets of our communities that are not very compliant, maybe because of their cultural beliefs or religious beliefs. We cannot say the significance in terms of numbers, but we know that these populations are there in Botswana and they are not confined to one place” (Table 2, Row 13–14).* Thus, interviews with study respondents did not reveal significant cultural norms impeding vaccine uptake among community members.

Reported vaccine hesitancy was a result of concerns about adverse events following immunization (AEFIs). This was a critical concern, especially because there was weak coordination of reporting and feedback on AEFIs, despite the establishment of an authority and committee mandated with this role. This hesitancy was observed across the population regardless of gender, age and other personal characteristics. Vaccine hesitancy was also believed to be due to negative beliefs, including mistrust, religious beliefs and conspiracy theories. Fear of the unknown scientific results, misconceptions and misinformation surrounding the efficacy and side effects of the vaccine led to mixed feelings about vaccine acceptance among the target population. Younger people were especially reported to have shown some hesitancy towards COVID-19 vaccines (Table 2, Row 15).

### 3.5 Gender, vulnerable populations & equity

#### 3.5.1 Gender mainstreaming

Interviews with participants for this study did not show that any specific approaches were used to ensure gender programming, equity and reaching the vulnerable population. One participant summed it up by saying, “*COVID-19 was not just affecting women or men. So, I do not think there needed to be a gender lens …”* (Table 2, Row 16).

Many health service delivery mechanisms in Botswana still adopt a general population approach—specific efforts to ensure equity are those focusing on improving access for those living in rural and remote settings and in some instances, to include the production of education materials in other languages. Conversations with a representative of people with disabilities indicated they were excluded from nearly all the forums or committees, including those conducting community mobilization activities (Table 2, Row 17). There was also limited understanding of gender mainstreaming in health care service delivery, where there is a general perception that there are no apparent gender differences in COVID-19 vaccines uptake due to gendered factors (Table 2, Row 19), (Table 2, Row 30). Thus, participants could not speak to any specific approaches that were adopted to ensure gender mainstreaming, nor did they perceive that this was a critical concern when it came to access and equity for available services (Table 2, Rows 20–21).

However, it was noted that in many of the established committees that coordinated and led the COVID-19 vaccine roll-out in the country, the majority were male-led. Only in direct service delivery of vaccinating the population were women indicated to have played a pivotal role where also most of the nursing staff in the country are women, who were therefore deployed for the provision of vaccines (Table 2, Row 22).

#### 3.5.2 Vulnerable population

At the same time, many at-risk persons in relation to age and existing co-morbidities were prioritized in the COVID-19 vaccine distribution and delivery. The priority populations included the frontline workers, especially the health professionals. This was also related to how vulnerabilities were interpreted and used to inform distribution and delivery to ensure access for all (Table 2, Row 23).

As previously indicated, it is generally entrenched that people living in hard-to-reach areas face vulnerabilities related to delivery logistics and access to COVID-19 vaccines. While some felt that “*we have a system which is working, it is very broad and fortunately you find it in very remote areas” (Table 2, Row 24),* others believed that logistics needed improvement to better facilitate vaccine access for residents of remote locations (Table 2, Row 25).

### 3.6 Stakeholder engagement

Botswana's health care delivery system is modeled after the principles of primary healthcare—and has always taken on a multi-sectoral approach to community participation and service delivery. Stakeholder engagement, like in most of the country's health care service delivery, was key to ensuring equitable distribution and delivery of the COVID-19 vaccines. This was captured well by one of the participants who stated that there was strong stakeholder engagement at all stages (Table 2, Row 26).

From the onset of the pandemic, key stakeholders were involved, through their existing structures and other platforms that were urgently mobilized. Stakeholder engagement during planning and implementation was considered key to ensuring the equitable distribution and delivery of the COVID-19 vaccines. Most of the study participants reported having experience of working with CSOs in the delivery of health services and appreciated the expertise that the CSOs brought on board to enhance the collaborative efforts of combating the COVID-19 pandemic. For sustainable emergency response, the government of Botswana was encouraged to continue working with stakeholders in planning for the pandemic well in advance to ensure effective delivery of health services when the pandemic occurs (Table 2, Rows 25–27).

Stakeholders such as CSOs, community leaders, politicians, the private sector and others played vital roles in the vaccine roll-out. This meant working deliberately to secure stakeholders' trust, understanding their hopes and concerns, and putting them into consideration while planning and implementing interventions with them to contribute significantly to the success of vaccine distribution and delivery. Participants also highlighted the importance of stakeholder coordination to ensure effective engagement (Table 2, Row 27).

Study participants further emphasized the need to ensure early engagement of all key stakeholders, ensuring that they are involved in the planning phase to ensure effective implementation of efforts. This proactive approach ensures alignment and reduces resistance during implementation (Table 2, Row 28).

#### 3.6.1 Challenges in stakeholder engagement

Despite its importance, engaging multiple stakeholders was reported to present several challenges. One significant issue is the complexity of coordinating various entities with different agendas and capabilities. Ensuring that all stakeholders are aligned and their resources are used efficiently requires co-planning and communication—establishing effective coordination and effective mechanisms to ensure feedback loops are always maintained. Another challenge is ensuring inclusivity and equity among stakeholders, particularly concerning gender representation. The need for equitable representation is highlighted by the view that people with disability are often excluded (Table 2, Row 31).

Achieving genuine participatory action from stakeholders is complex. Participants stress that engagement should extend beyond mere consultation and involve stakeholders in defining problems, designing interventions, and evaluating outcomes. The emphasis on “participatory action” from the beginning to the end underscores the need for a continuous and inclusive engagement process (Table 2, Rows 27–28).

### 3.7 Public private partnership

Public-private partnership (PPP) facilitated effective and efficient decentralization of vaccine distribution and service delivery, thus helping to improve equitable distribution and delivery of vaccines. In Botswana, this primarily involved engaging the private sector to support the transportation of vaccines, providing sites and facilities where vaccines could be administered.

The effectiveness of the PPP was barely realized, as some respondents felt that the discussion around PPP started post-COVID-19. PPP was explored as an opportunity to manufacture COVID-19 vaccines locally, indicating the effort made by the head of state to attract investors that could support local manufacturing of the vaccines. The readiness of Botswana to manufacture vaccines locally was met by mixed responses, with some respondents feeling that the country was ready, whereas other respondents felt that Botswana's population was too small, with a small vaccine market and therefore, lacked the ability to attract investors (Table 2, Row 32).

Participants underscored the importance of engaging the private sector, noting that the government cannot do it alone. Key strategies for leveraging private sector role and engagement revolved around harnessing private sector resources and expertise, establishing continuous collaboration through active committees, offering financial incentives such as tax reduction, addressing profit concerns, and integrating private sector contributions into broader community efforts.

## 4 Discussion

This study presented key lessons for health system strengthening, highlighting policy entry points and adaptations needed to improve equitable vaccine distribution and delivery in Botswana. Generally well organized through a clear coordinated multi-sectoral approach, this study highlights the interplay between coordination mechanisms, stakeholder participation and equity—identifying the opportunities that remain for addressing gaps, especially in gender mainstreaming, private sector engagement for equitable vaccine distribution and delivery. These findings reinforce what De pas et al. (33) highlighted as a critical need for context-specific approaches that recognizes the dynamic nature of the different settings in which vaccine distribution and delivery happen.

Botswana's distribution and delivery of COVID-19 vaccines was characterized by the setting up of national coordinating committees, collaboration with local and international non-governmental organizations, and strategic use of existing public and private health facilities. Innovative outreach methods such as workplace campaigns and drive-through services at strategic community spaces promoted distribution and delivery of the COVID-19 vaccines. Similar to observations made by Oversby et al. (2023) and Stan (2023), collaborative governance and flexible delivery models enhance vaccine accessibility and uptake in resource-limited settings (8, 34). Nonetheless, despite these enablers, Botswana's vaccination distribution and delivery was limited by similar factors experienced elsewhere, including shortage of transport and cold chain facility, limited human resources and pockets of vaccine hesitancy driven by misinformation and religious beliefs (4–6).

The country's approach to COVID-19 vaccine roll-out, as described by study participants, did not explicitly incorporate gender considerations. While some participants believed that a gender-specific approach was unnecessary due to the universal impact of COVID-19, this perception overlooked the nuanced ways in which gender influences health outcomes and access. Notably, vaccine coordination committees were described as male-dominated, contradicting international guidance that advocates for gender-balanced planning and representation to ensure equitable decision-making (35). It is worth noting that generally, gender mainstreaming in health services delivery in Botswana remains an area for strengthening—although the country has made significant strides in improving women's education and access to available health services, key structural issues that exclude women and other vulnerable groups persist (36). One of the areas of weakness highlighted in this report points to poverty, unemployment, and lack of economic independence among women in Botswana, which increases their vulnerability to HIV and limits their access to services more broadly. Although study participants perceived Botswana's immunization system as gender neutral in terms of services being readily available and accessible for all at various public facilities as well as community-based spaces, this general assumption masks entrenched structural and social barriers affecting women and other vulnerable people, such as people with disabilities. For instance, in Botswana, frontline healthcare workers are predominantly female—during the COVID-19 pandemic, when workloads increased, these women were more susceptible to work-related stress, burnout, and mental health disorders such as depression, compared to their male counterparts (37). It is important to systematically integrate gender in all prevention, treatment, and care strategies to ensure that the unique needs of women, men, girls, and other vulnerable populations are taken into consideration if we are to build just and equitable health systems. Studies have shown that during the COVID-19 pandemic, men experienced severe COVID-19 symptoms, hospitalization and deaths compared to women (38, 39).

This lack of gender mainstreaming and the reported exclusion of people with disabilities (PWDs) from Botswana's vaccine distribution planning, decision-making processes and delivery mechanisms also reflects a broader and persistent challenge in public health emergency responses. Across Africa and beyond, the marginalization of vulnerable groups with specific accessibility needs has generally been excluded or faced sub-optimal access to available health services (40, 41). More often than not, critical systemic gaps in health governance and emergency preparedness frameworks overlook or inadequately address the unique challenges faced by PWDs. Excluding PWDs in public health service delivery does not align with the Nation's Vision 2036 and directly contravenes principles of equity and human rights enshrined in global health agendas and the essential functions of public health (10, 11, 42). Such exclusion impairs the effectiveness of public health interventions when the health system fails to reach all segments of the population that also need services. Excluded from accessing services, information deprivation, and social stigma, PWDs often face compounded vulnerabilities during crises, increasing health disparities among country populations. The COVID-19 pandemic has thus highlighted the urgency for governments, practitioners, and all stakeholders to embed disability-inclusive policies within emergency preparedness and response mechanisms. This includes proactive identification of PWDs' needs, meaningful participation of disability representatives in planning structures, including technical working groups, and implementation of tailored interventions to ensure equitable accessibility. Addressing these gaps is integral to achieving universal health coverage and fostering resilient, inclusive health systems.

Similar to global and regional practices, the findings of this study reveal that Botswana's vaccine distribution and delivery strategy prioritized frontline healthcare workers, individuals with underlying health conditions, and the elderly (43–46). This approach has also been indicated as critical in public health, it reflects a sound risk-based strategy that prioritizes the efficient allocation of limited resources to reduce morbidity and mortality among the most vulnerable populations, which is also an essential component of building resilient health systems (47).

Measures to promote equity in vaccine distribution included targeted strategies for reaching hard-to-reach, remote populations. Although vaccines were delivered through static health facilities and temporary vaccination sites to enhance accessibility, findings from this study suggest that these initiatives were not consistently successful, as some participants indicated that logistical aspects required further refinement to adequately serve isolated communities. Challenges with transportation shortages and poor management of cold chains for vaccine storage emerged as persistent challenges in remote areas. These challenges are not unique to Botswana and have been reported in other African countries (48), where innovative solutions such as motorcycle-based delivery systems have been employed to navigate difficult terrains. In this context, leveraging the private sector for transportation and vaccine provision in remote sites proved essential in improving vaccine distribution in Botswana. Nonetheless, some participants expressed concern over delays in engaging the private sector during the initial stages of the pandemic response, underscoring the need for timely coordination. Moreover, participants emphasized the significance of coordinating stakeholders such as civil society, community leaders, and private enterprises to manage their potential interests and impact and to involve them in the initial deployment planning process. Such engagement of these entities is critical for addressing vaccine misinformation in communities, thus reducing vaccine hesitancy, thereby promoting equitable access, as evidenced in other African settings (49). Additionally, the concept of local vaccine production through public-private partnerships was explored, though opinions varied regarding Botswana's preparedness for such initiatives. While efforts have been made to attract investments in domestic vaccine manufacturing, concerns were raised about the country's small market size and its implications for investment viability. This reflects a continental challenge where only a few countries currently produce vaccines through collaborative public-private partnerships (50). This limitation undermines efforts towards timely and equitable vaccine distribution during health emergencies within the continent.

### 4.1 Study limitations

This study is not without limitations. The first limitation of the study is its focus on policymakers, excluding the implementers and community voices, which could have clarified the community's perceived barriers and facilitators that affected the uptake of the vaccines. While this omission does not take away the relevance of our findings to the public health system in Botswana and other settings with similar realities, future research should include community voices to ensure comprehensiveness of the research findings. The study employed purposive sampling, which may introduce some selection bias. This was mainly due to study time factors. However, the study findings' validation workshop greatly enhanced the quality of our findings, informed by relevant experts on the COVID-19 vaccine distribution and delivery in Botswana.

## 5 Recommendations

This study highlighted and emphasized the positive role of multisectoral partnerships in vaccine distribution and delivery. Most important, pointing to ensuring early or timely engagement of key stakeholders where effective public-private collaborations should be leveraged upon to expand reach, particularly in remote and hard-to-reach areas, and enhance system resilience for future emergencies.

There is an urgent need to ensure systematic and clear approaches are used to integrate gender-responsive approaches in vaccine planning, distribution and delivery to ensure equitable access. This includes ensuring gender-balanced representation in coordinating mechanisms or committees and tailoring interventions to address specific needs and barriers faced by women, men, and vulnerable groups like persons with disabilities, ensuring that women and those as risk of being excluded from access services when they need them, are adequately supported. Persons with disability should be engaged in meaningful ways during planning and service delivery to address any structural exclusion of these vulnerable groups.

Strengthening community trust and uptake of services by institutionalizing multi-actor coalitions and engagement, with clear roles that complement and align actors interests, would go a long way in addressing misinformation and vaccine hesitancy. Pockets of religious beliefs observed among minority groups, should be adquetly addressed and not generalized, as these have the potential to further exclude vulnerable groups among such communities, especially women and children.

## 6 Conclusion

The approach to COVID-19 vaccine distribution and delivery in Botswana showed coordination and collaboration across several sectors. While there were important achievements realized, there are opportunities for improvement in considering gender mainstreaming, identification and inclusion of vulnerable communities in planning and implementation, and early effective stakeholder engagement. Achieving equitable vaccine access involves not only logistical and infrastructural considerations, but also enhancing gender diversity and inclusivity in planning, coordination, and decision-making and implementation of strategies tailored to the needs of a wide range of vulnerable population groups.

## Data Availability

The raw data supporting the conclusions of this article will be made available by the authors, without undue reservation.
