# Supplementary material for: A qualitative analysis exploring barriers and enablers to distribution, delivery, and access to COVID-19 vaccines in Botswana
Source: Front Health Serv. 2025 Nov 18;5:1609056. doi: 10.3389/frhs.2025.1609056 (PMC12669139; doi:10.3389/frhs.2025.1609056)
Supplement: Supplementary file 1 [file Supplementaryfile1.docx]

**Supplement 1: Key Informant interview guide**

**Section 1: General information**

| Interview type: | Key Informant Interview (KII) |
| --- | --- |
| Country |  |
| Level of the interview: | *[MoH], [Partners]….* |
| Affiliation and role of interviewee: |  |
| Location of Interview: |  |
| Respondent number (ID): |  |
| Name of Interviewer: |  |
| Date of interview: |  |
| Duration of the interview | Starting time:  Ending Time:  Duration: /____/_____/ minutes |
| General observations/comments  e.g., interview cut short, noisy environment etc. |  |

**Section 2: Background information**

| No | Characteristics | Responses |
| --- | --- | --- |
| 1 | Age: |  |
| 2 | Sex: |  |
| 3 | Highest level of your educational qualification: |  |
| 4 | Brief description of his/her role in organization |  |
| 5 | Years of service in COVID-19 vaccination related works/vaccine/immunization related works |  |
| 6 | Years of service in management or leadership position in the public health system/management structure [current position] |  |

[No item should be left unanswered; say none if no comments. Use additional paper as needed].

Interviewer: __________________________

| **Qualitative topic guide to assess COVID-19 vaccines distribution and delivery** |
| --- |
| **Section 1: Introduction**   1. How familiar are you with the vaccine distribution and delivery mechanisms in Botswana particularly for COVID-19 vaccines? What is your role and responsibility in this?   **Section 2: Understanding Existing Mechanisms**   1. Can you describe Botswana’s current vaccine distribution and delivery mechanisms for COVID-19 vaccines? 2. What challenges or gaps have you observed in the existing vaccine distribution and delivery mechanisms in Botswana?   **Probe:** Especially concerning reaching out to vulnerable populations?   1. How do you see the availability of resources including manpower? 2. What does the supply chain look like? Are there any challenges that were encountered [**kindly spell them out in order of importance**]? 3. What is your opinion about the demand creation and surveillance work in Botswana with regard to vaccine sourcing, manufacturing & distribution? 4. Are there any efforts in Botswana to start vaccine manufacturing domestically and/or attract foreign investments to set up manufacturing companies for vaccines? 5. If there are manufacturing companies for vaccines set-up, which vaccines are being produced?   **Section 3: Vulnerable Populations and Equity**   1. Who, in your opinion, are the most vulnerable populations in Botswana concerning access to and administration of COVID-19 vaccines?   **Probe:** Are there vulnerable populations including refugees and other displaced populations, and people living with disabilities in Botswana?   1. Were these vulnerable populations reached and vaccinated for COVID-19 during the 2020, 2021 year when COVID-19 was at its peak? 2. What percentage of the vulnerable population received COVID-19 vaccines during these outreaches of vaccination for COVID? 3. What is your opinion regarding equity in vaccine distribution? 4. How do you think these vulnerable populations can be identified or reached effectively for vaccination?   **Section 4: Required Structures and Mechanisms**   1. What additional structures or mechanisms do you believe are necessary to ensure timely access to and administration of vaccines in Botswana, particularly for vulnerable populations? 2. Are there any successful models or practices that Botswana used or should adopt for improved vaccine distribution and delivery?   **Section 5: Stakeholder Involvement**   1. Which major stakeholders are engaged in a coordinated action plan including advocacy to address inequities in access to vaccines in your country? 2. How important is it to involve various stakeholders, such as government agencies, healthcare providers, community organizations, and international partners, in the improvement of vaccine distribution and delivery mechanisms? 3. What specific roles do you think these stakeholders should play in ensuring equitable vaccine distribution in Botswana?   **Probe:** How can non-governmental bodies, such as humanitarian organizations, contribute to addressing vaccine delivery challenges in Botswana?   1. Do you have experience or expertise in engaging civil society organizations and the private sector in public-private partnerships for healthcare initiatives in Botswana? Can you describe your experience? 2. What are the key advantages of public-private partnerships in improving vaccine delivery and access in Botswana? 3. Can you provide examples of successful public-private partnerships in the healthcare sector, particularly regarding vaccine distribution, that can serve as models for Botswana? 4. How can the private sector, including pharmaceutical companies and healthcare providers, be effectively engaged in supporting equitable vaccine distribution in Botswana?   **Probe:** What incentives or strategies can encourage private sector involvement in addressing vaccine access disparities**?**   1. What potential challenges or concerns do you foresee in engaging civil society organizations, non-governmental bodies, and the private sector in improving vaccine delivery in Botswana? 2. How can these challenges be mitigated or addressed to ensure the success of such collaborations?   **Section 6: Impact and Evaluation**   1. How would you assess the impact of improved vaccine distribution and delivery mechanisms on the overall public health situation in Botswana? 2. What key performance indicators or metrics should be used to evaluate the success of these mechanisms?   **Section 7: Recommendations**   1. Do you have any specific recommendations for policymakers and healthcare authorities in Botswana to enhance vaccine distribution and delivery mechanisms for COVID-19 vaccines, with a focus on vulnerable populations?   **Section 8: Additional Comments**   1. Is there any other information or insights you would like to share regarding this topic? |
